# Supplementary material for: Assessing Trail Running Biomechanics: A Comparative Analysis of the Reliability of StrydTM and GARMINRP Wearable Devices
Source: Sensors (Basel). 2024 Jun 1;24(11):3570. doi: 10.3390/s24113570 (PMC11175203; doi:10.3390/s24113570)
Supplement: Supplementary file 1 [file sensors-24-03570-s001.zip › sensors-2982468-supplementary.pdf]

sensors

MDPI

Article

Assessing Trail Running Biomechanics: A Comparative Analysis of the Reliability of Stryd™ and GARMINRP Wearable Devices

César Berzosa [1,2], Cristina Comeras-Chueca [1,2,*], Pablo Jesus Bascuas [1,2], Héctor Gutiérrez [1,2] and Ana Vanessa Bataller-Cervero [1,2]

[1] Faculty of Health Sciences, Universidad San Jorge, Autov. A-23 km 299, 50830 Villanueva de Gállego, Spain
[2] ValorA Research Group, Health Sciences Faculty, Universidad San Jorge, 50830 Villanueva de Gállego, Spain
* Correspondence: ccomeras@usj.es

Supplementary Material

In the Supplementary Materials of this research article, graphical representations of the statistical analyses conducted during the study have been included. These supplementary materials aim to provide readers with a more detailed and visual understanding of the data analysis process, supporting the central findings presented in the main manuscript. The graphs and charts included in this supplementary section serve as a valuable resource for those interested in a deeper exploration of the research outcomes and enhance the transparency and reproducibility of our study.

Intra-Device Reliability Analysis: GARMINRP

Bland-Altman Analysis

All the track

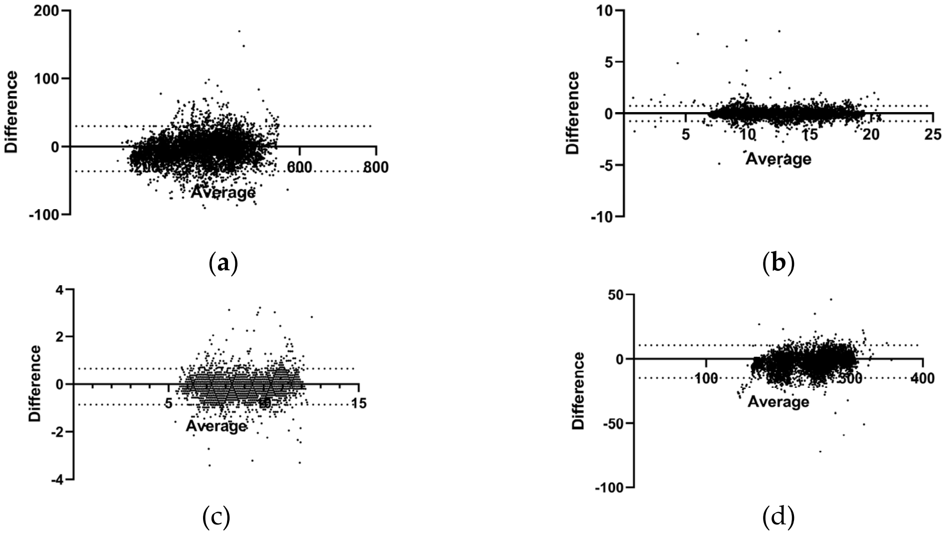

Figure S1. Bland–Altman plots of the rate of running in place as measured with (a) Power (W), (b) Speed (km/h), (c) Vertical Oscillation (cm), (d) Contact time (ms). The dashed lines represent the limits of agreement (LoA), while the solid lines depict bias.

Uphill

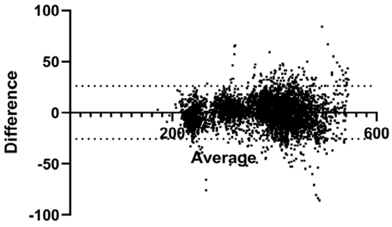

(**a**)

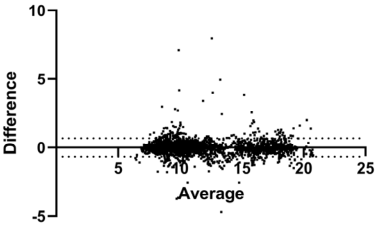

(**b**)

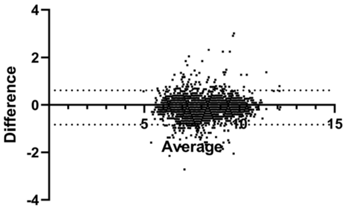

(c)

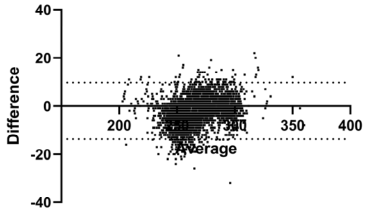

(d)

Figure S2. Bland–Altman plots of the rate of running in place as measured with (a) Power (W), (b) Speed (km/h), (c) Vertical Oscillation (cm), (d) Contact time (ms). The dashed lines represent the limits of agreement (LoA), while the solid lines depict bias.

Downhill

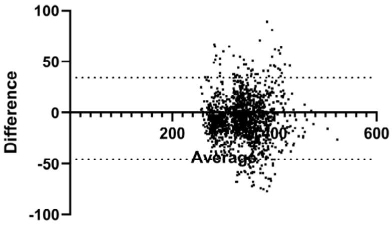

(**a**)

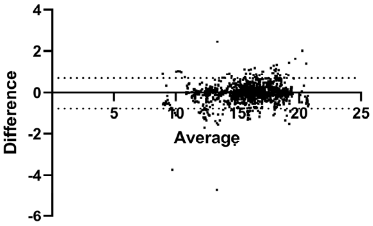

(**b**)

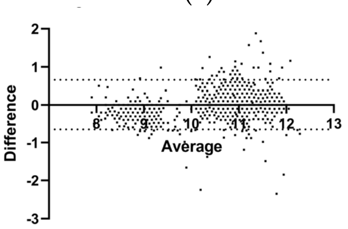

(c)

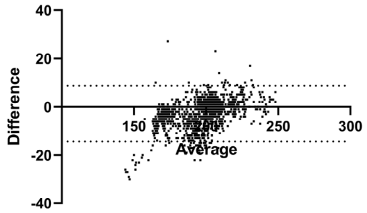

(d)

Figure S3. Bland–Altman plots of the rate of running in place as measured with (a) Power (W), (b) Speed (km/h), (c) Vertical Oscillation (cm), (d) Contact time (ms). The dashed lines represent the limits of agreement (LoA), while the solid lines depict bias.

Univariate Linear Regression Analysis: GARMINRP

All the track

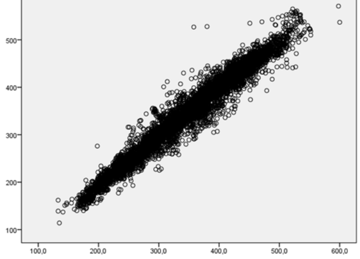

(**a**)

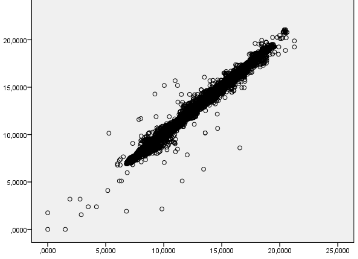

(**b**)

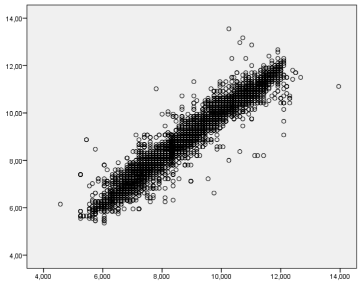

(c)

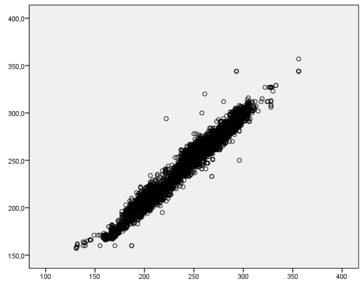

(d)

Figure S4. Univariate Linear Regression Analysis of (**a**) Power (W); (**b**) Speed (km/h); (**c**) Vertical Oscillation (cm); (**d**) Contact Time (ms).

Uphill

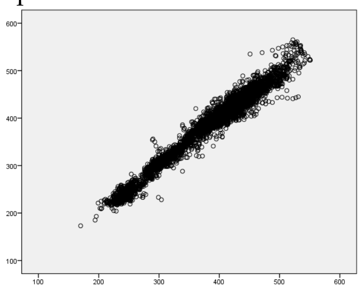

(**a**)

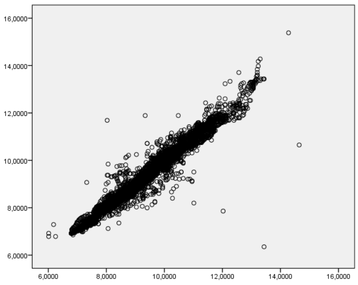

(**b**)

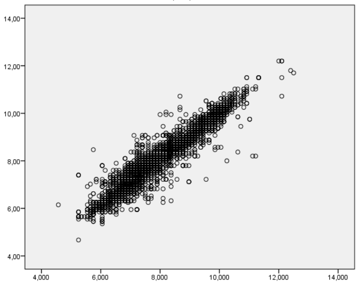

(c)

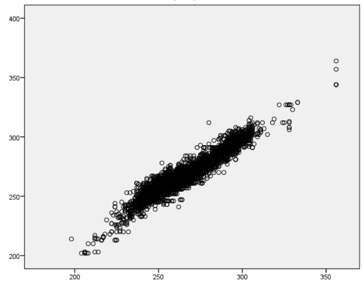

(d)

Figure S5. Univariate Linear Regression Analysis of (**a**) Power (W); (**b**) Speed (km/h); (**c**) Vertical Oscillation (cm); (**d**) Contact Time (ms).

Downhill

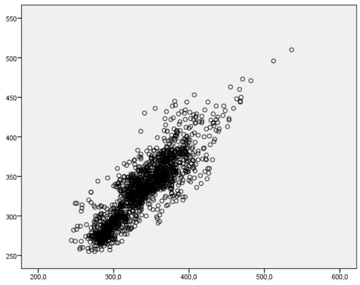

(**a**)

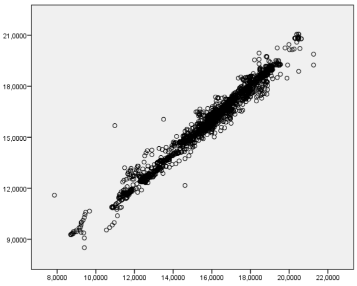

(**b**)

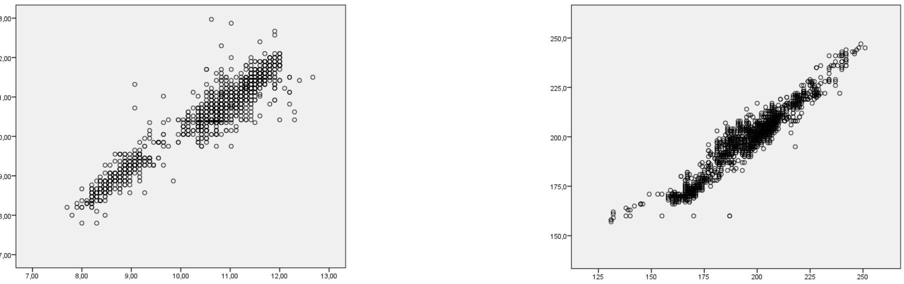

(c) (d)

Figure S6. Univariate Linear Regression Analysis of (**a**) Power (W); (**b**) Speed (km/h); (**c**) Vertical Oscillation (cm); (**d**) Contact Time (ms).

Intra-Device Reliability Analysis: Stryd™

Bland-Altman Analysis

All the track

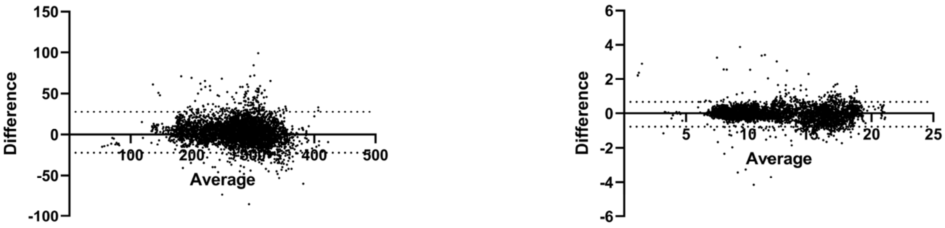

(**a**) (**b**)

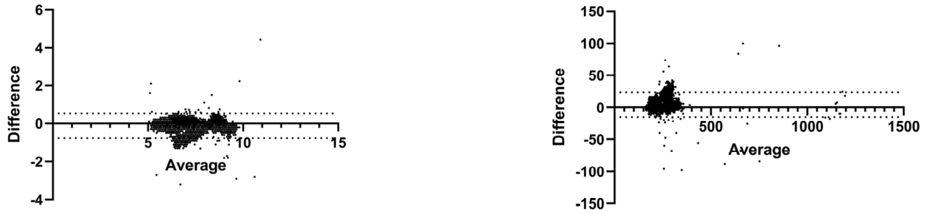

(c) (d)

Figure S7. Bland–Altman plots of the rate of running in place as measured with (a) Power (W), (b) Speed (km/h), (c) Vertical Oscillation (cm), (d) Contact time (ms). The dashed lines represent the limits of agreement (LoA), while the solid lines depict bias.

Uphill

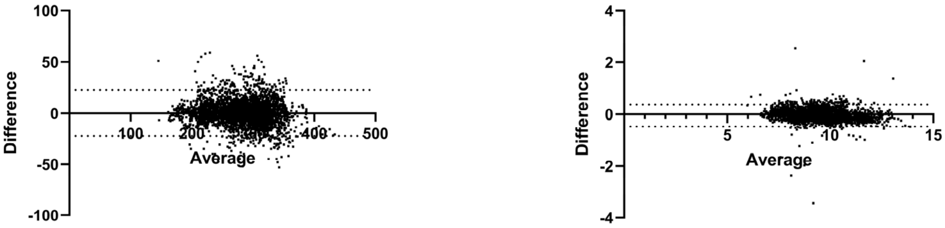

(**a**) (**b**)

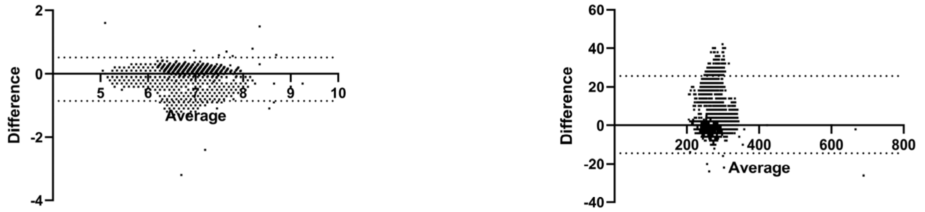

(c) (d)

Figure S8. Bland–Altman plots of the rate of running in place as measured with (a) Power (W), (b) Speed (km/h), (c) Vertical Oscillation (cm), (d) Contact time (ms). The dashed lines represent the limits of agreement (LoA), while the solid lines depict bias.

Downhill

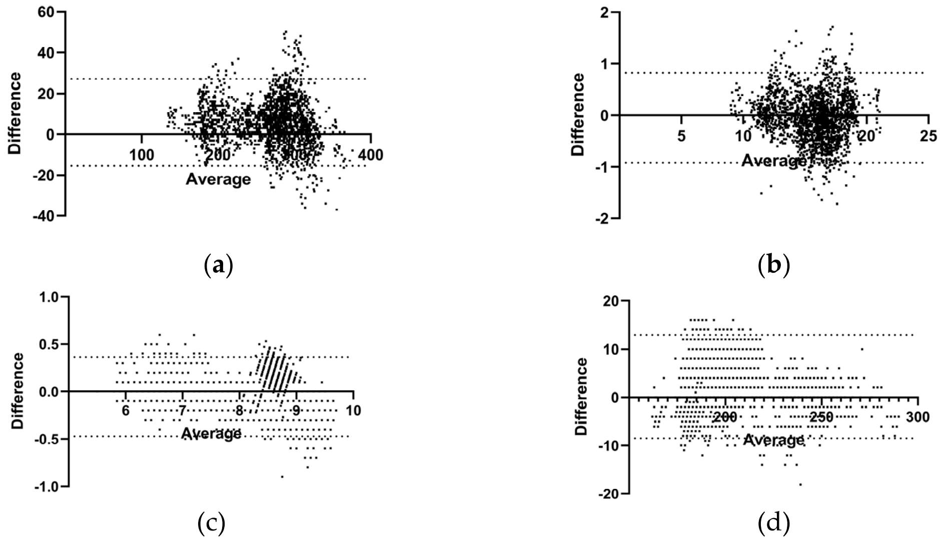

Figure S9. Bland–Altman plots of the rate of running in place as measured with (a) Power (W), (b) Speed (km/h), (c) Vertical Oscillation (cm), (d) Contact time (ms). The dashed lines represent the limits of agreement (LoA), while the solid lines depict bias.

Univariate Linear Regression Analysis: StrydTM

All the track

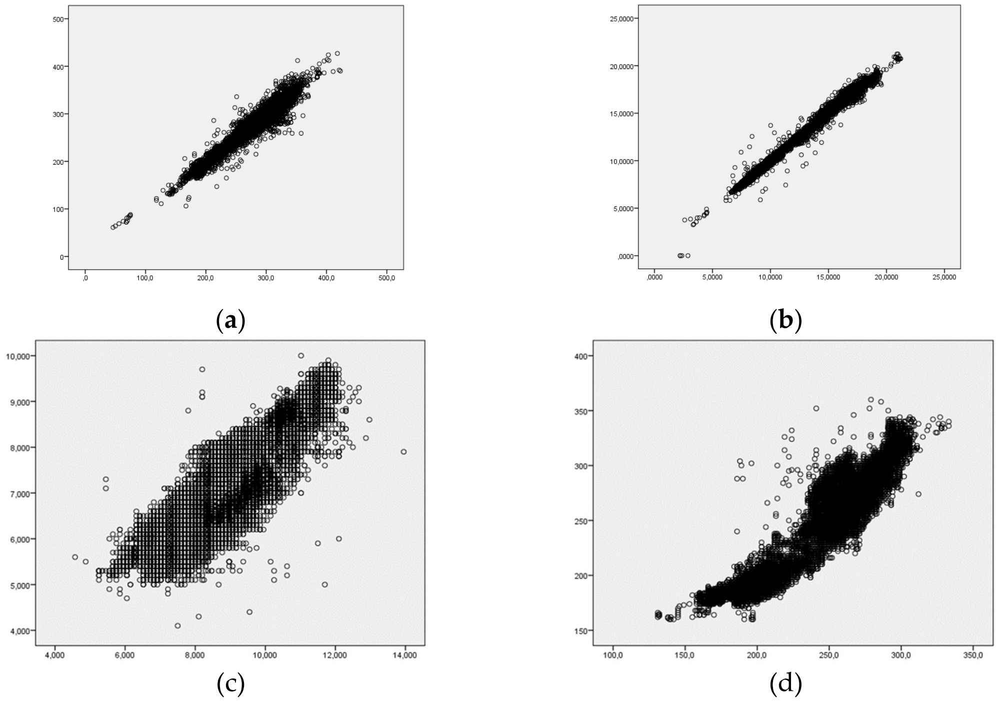

Figure S10. Univariate Linear Regression Analysis of (**a**) Power (W); (**b**) Speed (km/h); (**c**) Vertical Oscillation (cm); (**d**) Contact Time (ms).

Uphill

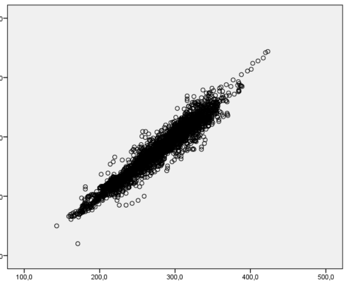

(**a**)

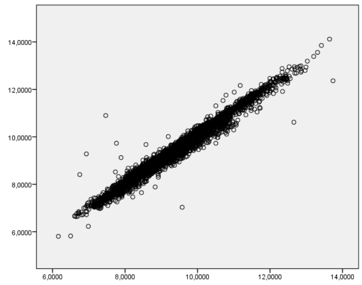

(**b**)

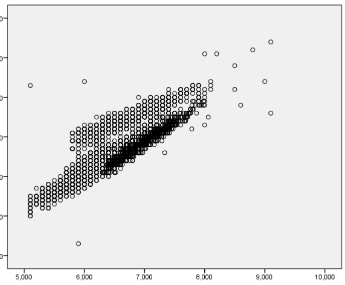

(c)

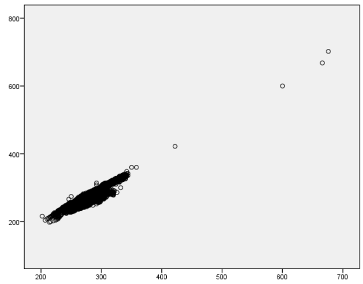

(d)

Figure S11. Univariate Linear Regression Analysis of (**a**) Power (W); (**b**) Speed (km/h); (**c**) Vertical Oscillation (cm); (**d**) Contact Time (ms).

Downhill

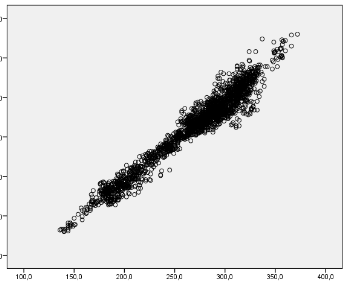

(**a**)

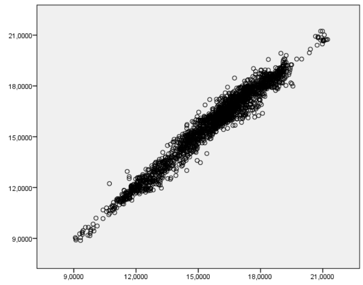

(**b**)

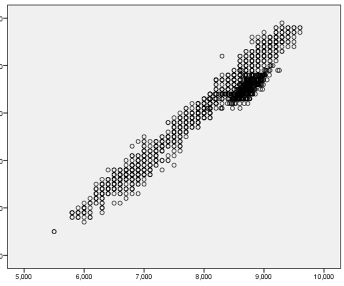

(c)

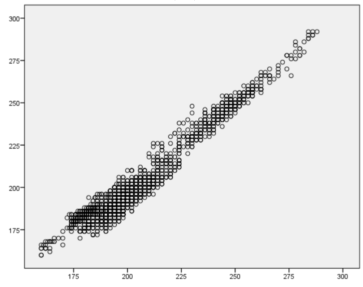

(d)

Figure S12. Univariate Linear Regression Analysis of (**a**) Power (W); (**b**) Speed (km/h); (**c**) Vertical Oscillation (cm); (**d**) Contact Time (ms).

Inter-Device Agreement

Bland-Altman Analysis

All the track

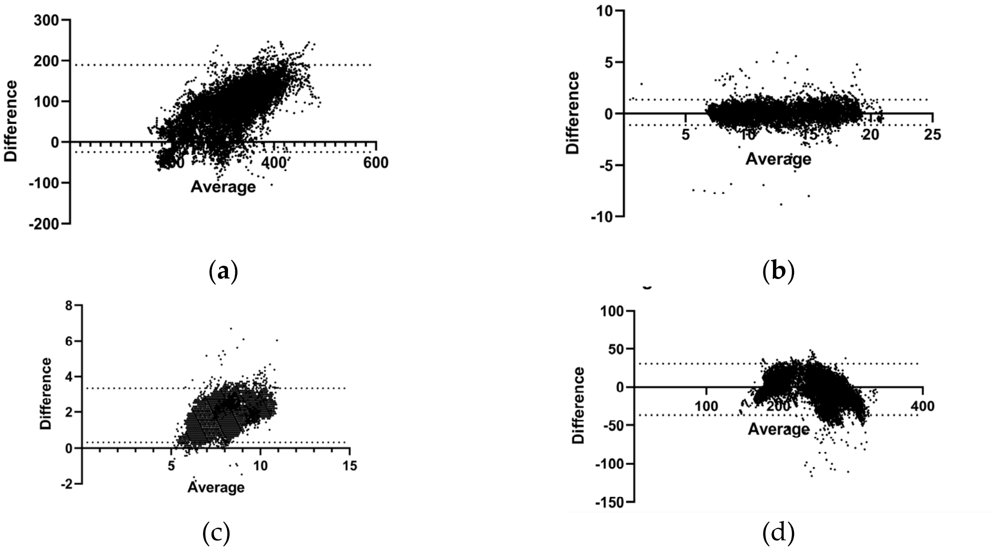

Figure S13. Bland–Altman plots of the rate of running in place as measured with (a) Power (W), (b) Speed (km/h), (c) Vertical Oscillation (cm), (d) Contact time (ms). The dashed lines represent the limits of agreement (LoA), while the solid lines depict bias.

Uphill

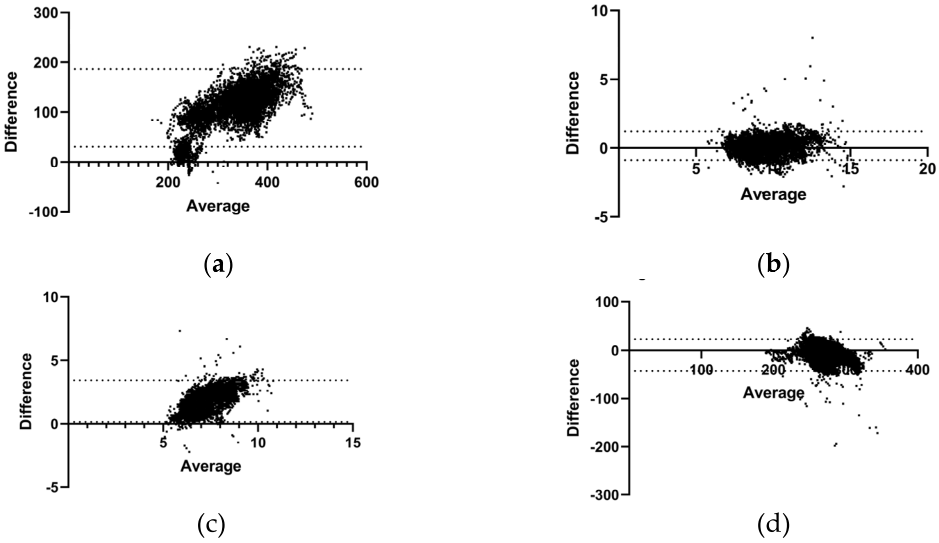

Figure S14. Bland–Altman plots of the rate of running in place as measured with (a) Power (W), (b) Speed (km/h), (c) Vertical Oscillation (cm), (d) Contact time (ms). The dashed lines represent the limits of agreement (LoA), while the solid lines depict bias.

Downhill

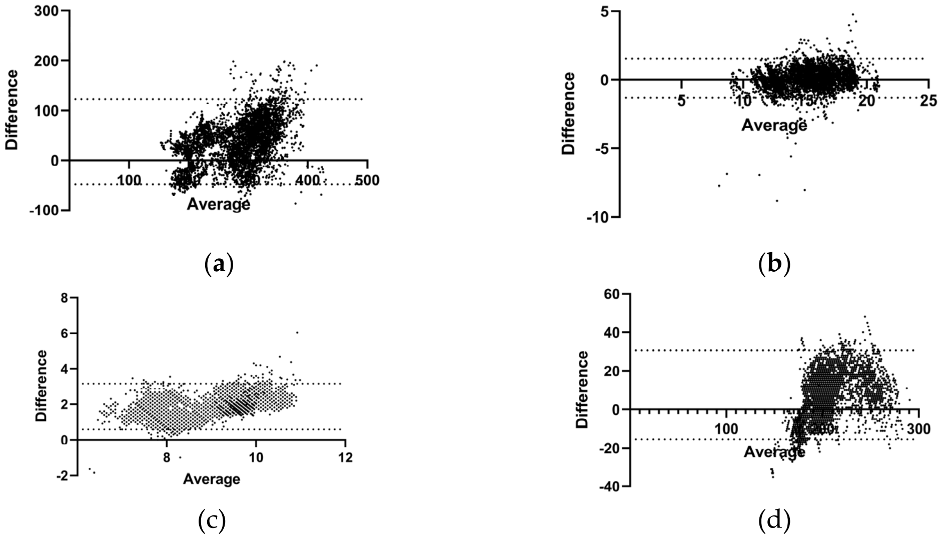

(**a**) (**b**)

(c) (d)

Figure S15. Bland–Altman plots of the rate of running in place as measured with (a) Power (W), (b) Speed (km/h), (c) Vertical Oscillation (cm), (d) Contact time (ms). The dashed lines represent the limits of agreement (LoA), while the solid lines depict bias.

Univariate Linear Regression Analysis

All the track

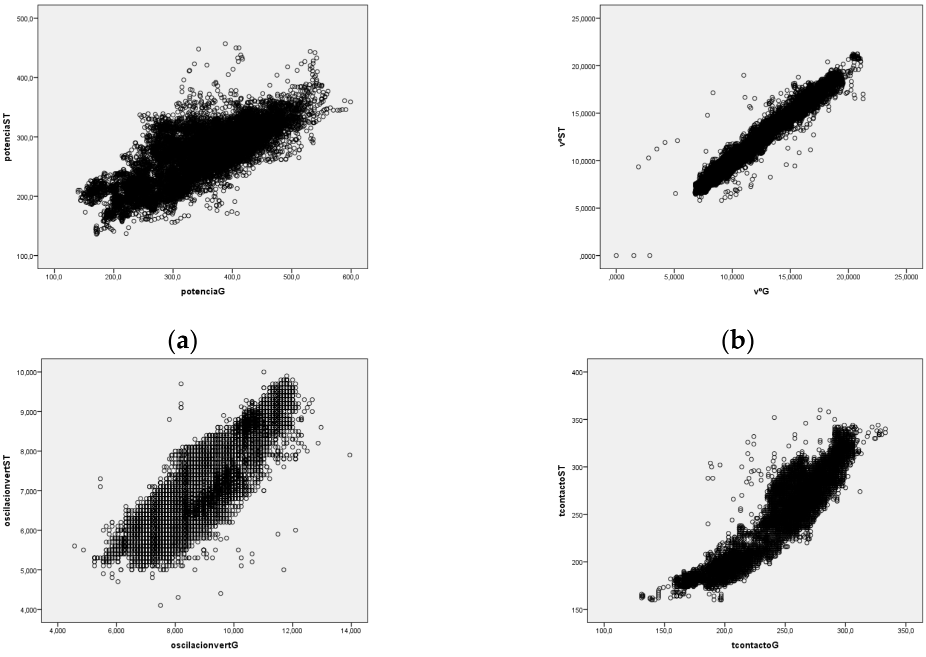

(**a**) (**b**)

(c) (d)

Figure S16. Univariate Linear Regression Analysis of (**a**) Power (W); (**b**) Speed (km/h); (**c**) Vertical Oscillation (cm); (**d**) Contact Time (ms). The y-axis represents Garmin$_{RP}$ measurements, while the x-axis represents Stryd™ measurements.

Uphill

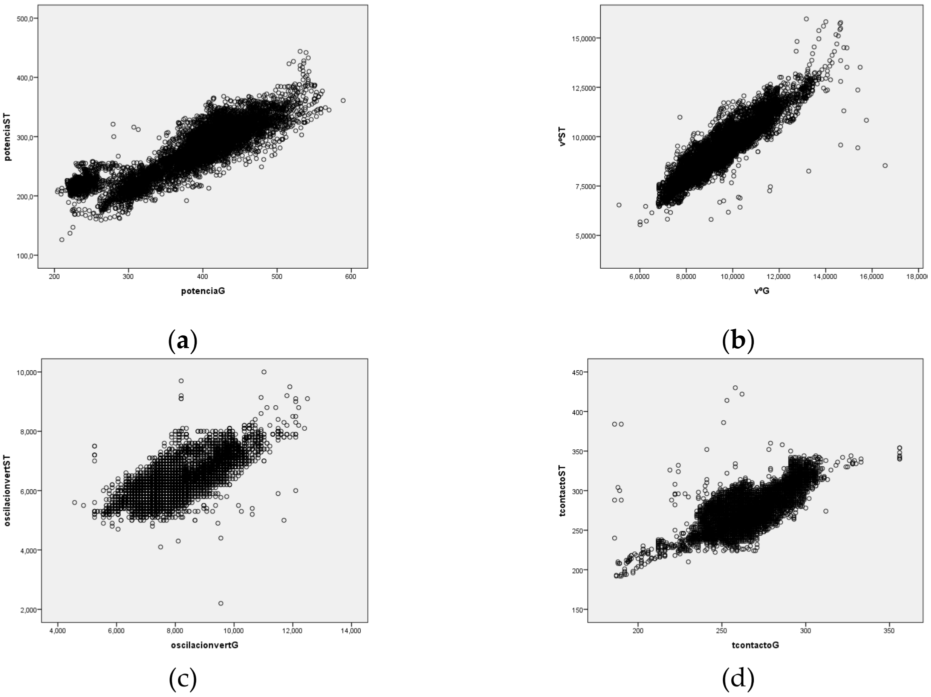

Figure S17. Univariate Linear Regression Analysis of (**a**) Power (W); (**b**) Speed (km/h); (**c**) Vertical Oscillation (cm); (**d**) Contact Time (ms). The y-axis represents GarminRP measurements, while the x-axis represents Stryd™ measurements.

Downhill

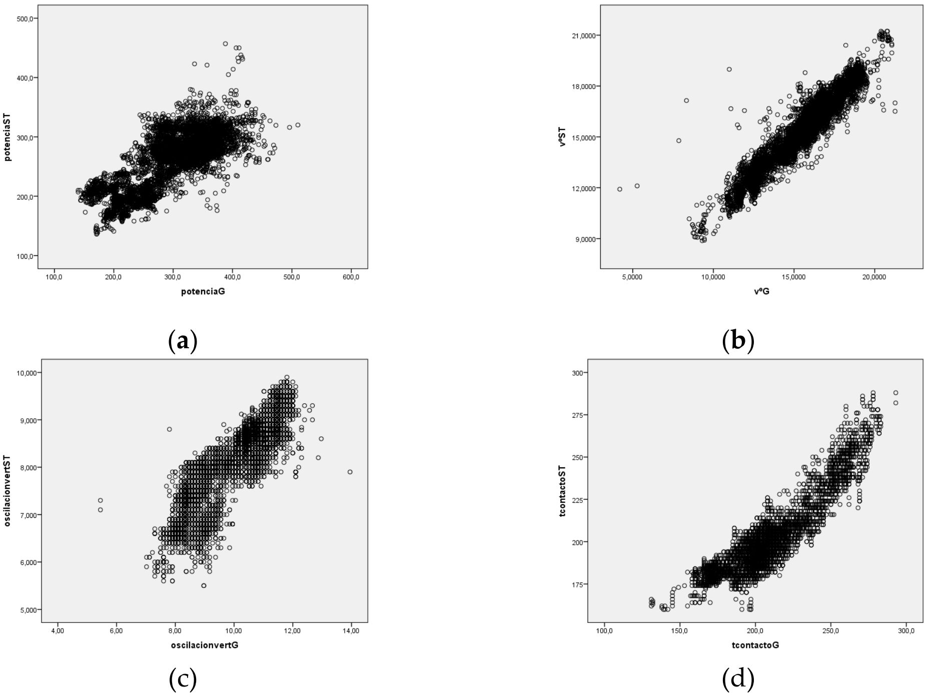

Figure S18. Univariate Linear Regression Analysis of (**a**) Power (W); (**b**) Speed (km/h); (**c**) Vertical Oscillation (cm); (**d**) Contact Time (ms). The y-axis represents GarminRP measurements, while the x-axis represents Stryd™ measurements.
